# Determining Thermophysical Parameters of Cryopreserved Articular Cartilage Using Evolutionary Algorithms and Experimental Data

**DOI:** 10.3390/ma17235703

**Published:** 2024-11-21

**Authors:** Alicja Piasecka-Belkhayat, Anna Skorupa, Marek Paruch

**Affiliations:** Department of Computational Mechanics and Engineering, Silesian University of Technology, Konarskiego 18A, 44-100 Gliwice, Poland; alicja.piasecka-belkhayat@polsl.pl (A.P.-B.); marek.paruch@polsl.pl (M.P.)

**Keywords:** bioheat and mass transfer, cryopreservation, evolutionary algorithm, inverse problem, liquidus tracking method, numerical methods

## Abstract

Cryopreservation is the process of freezing and storing biological cells and tissues with the purpose of preserving their essential physiological properties after re-warming. The process is applied primarily in medicine in the cryopreservation of cells and tissues, for example stem cells, or articular cartilage. The cryopreservation of articular cartilage has a crucial clinical application because that tissue can be used for reconstruction and repair of damaged joints. This article concerns the identification of the thermophysical parameters of cryopreserved articular cartilage. Initially, the direct problem was formulated in which heat and mass transfer were analyzed by applying the finite difference method. After that, at the stage of inverse problem investigations, an evolutionary algorithm coupled with the finite difference method was used. The identification of the thermophysical parameters was carried out on the basis of experimental data on the concentration of the cryoprotectant. In the last part, this article presents the results of numerical analysis for both the direct and inverse problems. Comparing the results for the direct problem, in which the thermophysical parameters are taken from the literature, with the experimental data, we obtained a relative error between 0.06% and 15.83%. After solving the inverse problem, modified values for the thermophysical parameters were proposed.

## 1. Introduction

Cryopreservation is a significant field of cryobiology that is constantly evolving. Cryopreservation refers to the freezing of animal and human cells and tissues, such as oocytes, embryos, spermatozoa, hepatocytes and others. The research about cryopreservation also explores the storage of organs for transplantation and stem cells or articular cartilage at low temperatures. It is important in the development of tissue engineering, which is an opportunity for soft tissue regeneration and the treatment of many diseases. It is also noteworthy that the cryopreservation of articular cartilage has a crucial clinical application because that tissue can be used for reconstruction and repair of damaged joints.

The aim of cryopreservation is to maintain cells or tissues with no significant impact on their essential functions, for example, viability and mechanical properties. Cryopreservation involves initially slowing down biological activity by cooling a sample to sub-zero temperatures (such as approximately −80 °C or −196 °C) and then reviving it by reheating to physiological temperature [1,2,3].

It is essential to avoid cell damage during this process. This is caused by biophysical changes, such as cell dehydration and the formation of intracellular and extracellular ice crystals [4]. To protect the cryopreserved biological material, a proper method can be used to optimize the cooling/warming rate, thereby controlling the transport of water across cell membranes as well as the intracellular water freezing processes. Another approach to prevent cell damage is the use of cryoprotectants (CPAs), such as glycerol (GLY) or dimethyl sulfoxide (DMSO) [3,5,6].

When using CPAs, care should be taken to select the right concentration, as too high of a concentration can lead to a slowing down of the heat transfer process due to reduced thermal conductivity and can also cause osmotic stress in cells or cytotoxicity. The concentration of the cryoprotectant also has a significant effect on the phase transformations occurring during the cryopreservation process. A high concentration of CPA reduces the freezing temperature of the cellular solution. In contrast, a low concentration of cryoprotectant does not provide sufficient protection of cells or tissues against low-temperature damage but protects against, for example, cell cytotoxicity. Successful cryopreservation therefore requires finding an optimal concentration that provides sufficient tissue and cell protection.

Cryopreservation can be carried out using the most common methods, slow freezing or vitrification, depending on the rate of cooling and concentration of cryoprotectant (CPA). Slow freezing is performed at a low cooling rate and a low concentration of CPA. Vitrification, where the liquid phase is transformed into the vitreous phase, involves a high rate of cooling and a high CPA concentration [1,2]. Other cryopreservation techniques include the “liquidus tracking” (LT) method. In this method, the biological specimen is immersed in a CPA solution with adjustable temperature and concentration. CPA molecules enter the cellular space of the samples concerned. This process is induced by molecular diffusion and osmotic transport because, when the cryoprotectant is in the extracellular matrix, the cells seek to equalize the pressure difference. This results in the exchange of components across the cell membrane and the entry of CPA into the cellular solution. This leads to a change in the freezing or melting point, while the solution’s thermodynamic conditions “track” the line of liquidus for the entire system under consideration. If the procedure is performed well, no ice crystallization will occur, and the biological sample will not be exposed to CPA-induced cytotoxicity [7].

Cryopreservation is a complex process, and preparing its multi-scale model requires knowledge not only of mechanics but also of biology and chemistry. During the cryopreservation process, transport phenomena mainly occur. First, heat transfer is a combination of convection and conduction. Thermal processes are most commonly described by the Fourier, Pennes, Cattaneo–Vernotte or dual-phase lag equations [2,8,9,10,11]. The phase changes associated with ice crystallization should also be taken into account when constructing a heat transport model [2].

During the cryopreservation process, mass transfer takes place in addition to heat transfer. It is induced by the molecular motion that takes place during the diffusion of a condensed cryoprotectant solution into the extracellular space. The convection–diffusion equation is used to characterize the phenomenon thus defined [12,13]. If advection is ignored, Fick’s law is applied to the mathematical description of particle diffusion [11,14].

In micro-scale, mass transfer is analyzed as a process of osmotic transport associated with changes in cell volume due to CPA and water molecules’ exchange between an extracellular and an intracellular solution. To determine these values, one can apply the Kedem–Katchalsky equations or the two-parameter formalism concept [15].

Similar analyses of heat and mass transfer have previously been presented in the literature [11,16,17]. Unfortunately, the numerical simulation results obtained were at variance with the experimental results, which may have been due to incorrectly chosen thermophysical parameters obtained experimentally. Therefore, it was decided to solve an inverse problem to estimate these parameters and compare them with the values found in the literature.

In this paper, the direct problem is defined as the axisymmetric heat and mass transfer in an articular cartilage sample during the cryopreservation process. The heat transfer occurring in the biological tissue was characterized using the Fourier equation, while the mass transport was described by an equation using Fick’s law. Osmotic transport was excluded from the analysis. The model presented here uses the LT protocol proposed by Pegg et al. [18]. In accordance with the LT protocol, the sample under study was placed in a chamber which was filled with bath solution. The computer-controlled temperature of the bath solution depends on the exposure time of the sample to the solution. Analogous to Pegg et al. [18], an aqueous solution of DMSO (CPA) was applied as a bath of solution in the simulation. The boundary and initial conditions that complement the mathematical model are in accordance with the LT protocol. The direct problem was solved using the finite difference method.

The inverse problem discussed in this article involves the simultaneous identification of three thermophysical parameters of biological tissue, i.e., thermal conductivity, density and specific heat capacity. An evolutionary algorithm (EA) is used to solve the identification problem formulated in this way [19,20,21].

## 2. Materials and Methods

**Figure 1:**
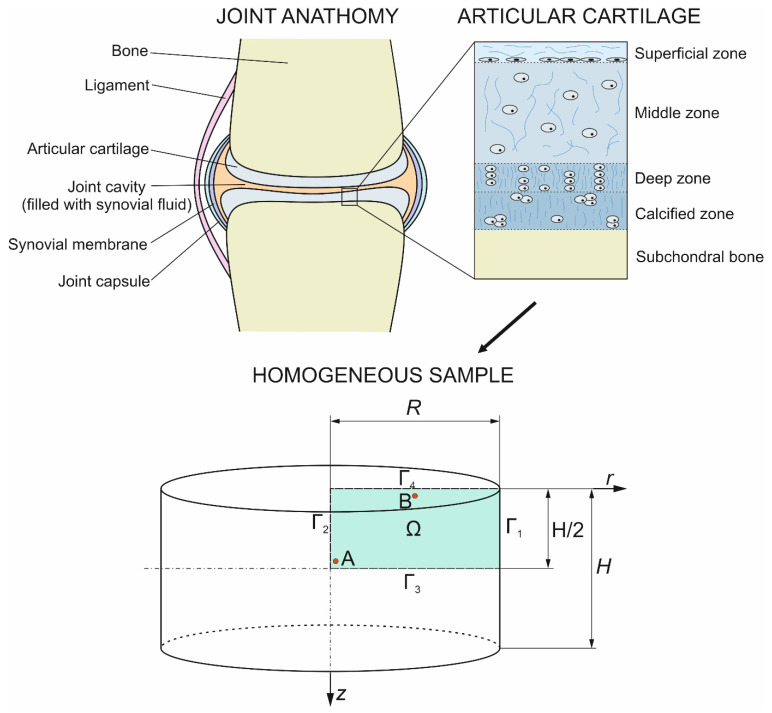
Geometry considered.

The numerical analysis was carried out for a two-dimensional model (axially symmetrical). Figure 1 shows the geometry of the analyzed problem and the visualization of the articular cartilage microstructure, including a scheme of the joint structure. In addition, two reference points (A and B with coordinates *r* = 0.00005 m, *z* = 0.000475 m and *r* = 0.00145 m, *z* = 0.000025 m, respectively) are marked on the diagram of the computational domain (Ω), where the simulation results are calculated.

### 2.1. Direct Problem

#### 2.1.1. Heat Transfer Model

To analyze the temperature distribution in biological tissue during cryopreservation, one can use the energy equation proposed by Jean Baptiste Joseph Fourier [11,22]:

(1) $$c\rho \frac{\partial T\left(X,t\right)}{\partial t}\left(X,t\right)=\nabla \left(\lambda \nabla T\left(X,t\right)\right)+Q\left(X,t\right)$$

where *T* is the temperature, *Q* is the heat source, *X* refers to the coordinate system, *t* is the time and *c*, ρ and λ represent the thermophysical parameters such as the specific heat capacity, the density and the thermal conductivity, respectively. Please note that articular cartilages do not have blood or lymphatic vessels; therefore, the heat source *Q* is neglected in further considerations. In addition, phase transformations are omitted because the LT protocol is applied [16,23].

Having studied the geometry of the sample (cf. Figure 1), the heat transfer equation can be formulated as follows [16,23]:

(2) $$c\rho \frac{\partial T(r,\;z,\;t)}{\partial t}=\frac{1}{r}\frac{\partial }{\partial r}\left(\lambda r\frac{\partial T(r,\;z,\;t)}{\partial r}\right)+\frac{\partial }{\partial z}\left(\lambda \frac{\partial T(r,z,t)}{\partial z}\right)$$

where *r* and *z* are the cylindrical coordinates.

The heat transfer equation (Equation (2)) was completed with an initial condition [16,23]:

(3) $$\begin{array}{cc} t=0: & T(r,z,0)=T_{0} \end{array}$$

and Neuman’s and Robin’s boundary conditions [16,23]:

(4) $$\left\{\begin{array}{cc} \Gamma_{1}\;\mathrm{and}\;\Gamma_{4}: & -n\lambda \cdot \nabla T=\alpha \left[T\left(r,z,t\right)-T_{bath}\right]\\ \Gamma_{2}\;\mathrm{and}\;\Gamma_{3}: & -n\lambda \cdot \nabla T=0 \end{array}\right.$$

where *T*_0_ is the initial temperature, **n** is the normal vector to the boundary and α is the natural convection heat transfer coefficient. *T_bath_* is the temperature of the surrounding medium (a bath solution).

#### 2.1.2. Mass Transfer Model

The mass transfer, which is weakly coupled to the thermal processes, can be described by the convection–diffusion equation [12,23]:

(5) $$\frac{\partial c_{d}\left(X,t\right)}{\partial t}=\nabla \left[D\left(T\right)\nabla c_{d}\left(X,t\right)\right]-\nabla \cdot \left(uc_{d}\left(X,t\right)\right)+R_{s}$$

where *c_d_* is the molar concentration, *D* is the molecular diffusion coefficient, **u** is the flow velocity field, *R_s_* is the source component associated with the occurrence of chemical reactions (in our case, the chemical equilibration is maintained, therefore *R_s_* = 0). The subscript *d* denotes the DMSO, which is a cryoprotectant in the mixture. As the biological sample is immersed in the solution during the process, it is assumed that the convection phenomenon is neglected (**u** = 0). While analyzing only the diffusion phenomenon, it can be said that the basis of the mass transfer equation is Fick’s second law [16].

In a cylindrical system, Equation (5) is expressed as follows [16]:

(6) $$\frac{\partial c_{d}\left(r,z,t\right)}{\partial t}=\frac{1}{r}\frac{\partial }{\partial r}\left(D\left(T\right)r\frac{\partial c_{d}(r,z,t)}{\partial r}\right)+\frac{\partial }{\partial z}\left(D\left(T\right)\frac{\partial c_{d}(r,z,t)}{\partial z}\right)$$

The diffusion coefficient can be determined using the Einstein–Stokes equation [16]:

(7) $$D\left(T\right)=\frac{k_{B}T}{6\pi r_{s}\mu }$$

where *k_B_* is the Boltzmann constant (*k_B_* = 1.38 × 10^−23^ J·K^−1^), *r_s_* is the spherical particle radius and μ is the dynamic viscosity.

In the model describing the mass transfer, the following initial and boundary conditions were included [16]:

(8) $$\left\{\begin{array}{cc} t=0: & c_{d}(r,z,0)=c_{0}\\ \Gamma_{1}\;\mathrm{and}\;\Gamma_{4}: & c_{d}\left(r,z,t\right)=0.9c_{bath}\\ \Gamma_{2}\;\mathrm{and}\;\Gamma_{3}: & -n\cdot D\left(T\right)\nabla c_{d}=0 \end{array}\right.$$

where *c*_0_ is the initial concentration, and *c_bath_* is the concentration of the surrounding media (bath solution). Adding a factor of 0.9 simulates the mass transfer between the sample and the bath solution.

### 2.2. Numerical Model

In this paper, an explicit scheme of the finite difference method (FDM) was used to create the numerical model [24,25].

The constant time step is introduced Δ*t* = *t ^f−^*^1^−*t ^f^*:

(9) $$\begin{array}{c} t^{0}<t^{1}<\ldots <t^{f-2}<t^{f-1}<t^{f}<\ldots <t^{F}<\infty \end{array}$$

**Figure 2:**
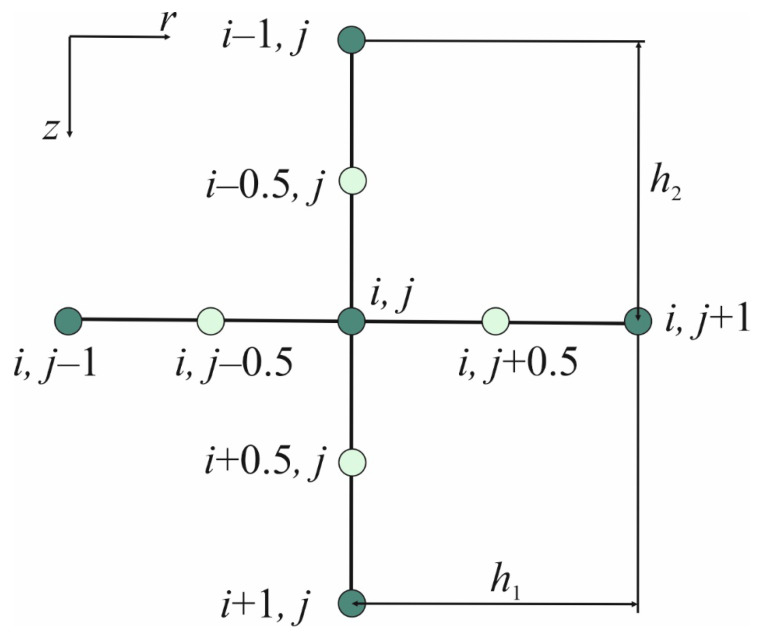
Five-point star.

The computational domain was discretized with the aid of a regular mesh using the five-point star concept as shown in Figure 2. To better approximate the boundary conditions, the boundary nodes were moved from the boundary by a distance of 0.5*h*_1_ and 0.5*h*_2_ [24].

Applying the FDM in the differential equation, one introduces differential quotients. The time derivative in the heat transfer equation is given by the following formula [17,25]:

(10) $${\left(\frac{\partial T}{\partial t}\right)}_{i,j}^{f}=\frac{T_{i,j}^{f}-T_{i,j}^{f-1}}{\Delta t}$$

and the derivatives on the right-hand side of Equation (2) in the internal nodes can be estimated as follows [17,25]:

(11) $$\begin{array}{c} {\left[\lambda \nabla^{2}T\right]}_{i,j}^{f-1}=\frac{1}{r_{i,j}}\frac{1}{h_{1}}\left[{\left(r_{i,j+0.5}\lambda \frac{\partial T}{\partial r}\right)}_{i,j+0.5}^{f-1}-{\left(r_{i,j-0.5}\lambda \frac{\partial T}{\partial r}\right)}_{i,j-0.5}^{f-1}\right]\\ +\frac{1}{h_{2}}\left[\lambda {\left(\frac{\partial T}{\partial z}\right)}_{i+0.5,j}^{f-1}-\lambda {\left(\frac{\partial T}{\partial z}\right)}_{i-0.5,j}^{f-1}\right] \end{array}$$

where *i* = 2, 3, …, *n* − 1 and *j* = 2, 3, …, *m* − 1; *n* and *m* are the number of nodes; *r_i,j_* is the radial coordinate of the node (*i*, *j*); and *h*_1_ and *h*_2_ are the mesh steps in the *r*- and *z*-direction, respectively.

The respective differential quotients are formulated as follows [17,25]:

(12) $$\begin{array}{l} \begin{array}{c} {\left(r\lambda \frac{\partial T}{\partial r}\right)}_{i,j+0.5}^{f-1}=r_{i,j+0.5}\;\lambda \frac{T_{i,j+1}^{f-1}-T_{i,j}^{f-1}}{h_{1}}=\left(r_{i,j}+\frac{h_{1}}{2}\right)\frac{T_{i,j+1}^{f-1}-T_{i,j}^{f-1}}{R_{i,j+1}} \end{array}\\ \begin{array}{c} \begin{array}{c} {\left(r\lambda \frac{\partial T}{\partial r}\right)}_{i,j-0.5}^{f-1}=r_{i,j-0.5}\;\lambda \frac{T_{i,j}^{f-1}-T_{i,j-1}^{f-1}}{h_{1}}=\left(r_{i,j}-\frac{h_{1}}{2}\right)\frac{T_{i,j}^{f-1}-T_{i,j-1}^{f-1}}{R_{i,j-1}} \end{array} \end{array}\\ {\left(\lambda \frac{\partial T}{\partial z}\right)}_{i+0.5,j}^{f-1}=\lambda \frac{T_{i+1,j}^{f-1}-T_{i,j}^{f-1}}{h_{2}}=\frac{T_{i+1,j}^{f-1}-T_{i,j}^{f-1}}{R_{i+1,j}}\\ {\left(\lambda \frac{\partial T}{\partial z}\right)}_{i-0.5,j}^{f-1}=\lambda \frac{T_{i,j}^{f-1}-T_{i-1,j}^{f-1}}{h_{2}}=\frac{T_{i,j}^{f-1}-T_{i-1,j}^{f-1}}{R_{i-1,j}} \end{array}$$

where *R* is the thermal resistance.

Finally, the following formula is obtained [17]:

(13) $$T_{i,j}^{f}=T_{i,j}^{f-1}+\frac{\Delta t}{c\rho }\left.\left[\left(\sum_{a=1}^{4}\frac{\Phi_{e}}{R_{e}}\left(T_{e}^{f-1}-T_{i,j}^{f-1}\right)\right)\right.\right]$$

where the individual *a* corresponds to *e* = {(*i*, *j* + 1); (*i*, *j* − 1); (*i* + 1, *j*); (*i* − 1, *j*)} and Φ*_e_* is the shape function:

(14) $$\begin{array}{ccc} \Phi_{i,j-1}=\frac{r_{i,j}-0.5h_{1}}{r_{i,j}h_{1}}, & \Phi_{i,j+1}=\frac{r_{i,j}+0.5h_{1}}{r_{i,j}h_{1}}, & \Phi_{i-1,j}=\Phi_{i+1,j}=\frac{1}{h_{2}} \end{array}$$

Considerations for temperatures at boundary nodes are made in a similar procedure and will not be discussed here—a detailed description can be found in the literature [17,24].

Analogously, a numerical model is formulated for the mass transfer phenomenon. The time derivative on the left-hand side of Equation (6) is of the following form [17]:

(15) $${\left(\frac{\partial c_{d}}{\partial t}\right)}_{i,j}^{f}=\frac{{\left(c_{d}\right)}_{i,j}^{f}-{\left(c_{d}\right)}_{i,j}^{f-1}}{\Delta t}$$

while the derivative on the right-hand side in the internal nodes is as follows [17]:

(16) $$\begin{array}{c} {\left[D\left(T\right)\nabla c_{d}\right]}_{i,j}^{f-1}=\frac{1}{r_{i,j}}\frac{1}{h_{1}}\left[D\left(T_{i,j+0.5}^{f-1}\right)r_{i,j+0.5}{\left(\frac{\partial c_{d}}{\partial r}\right)}_{i,j+0.5}^{f-1}\left.-D\left(T_{i,j-0.5}^{f-1}\right)r_{i,j-0.5}{\left(\frac{\partial c_{d}}{\partial r}\right)}_{i,j-0.5}^{f-1}\right]\right.\\ +\frac{1}{h_{2}}\left[D\left(T_{i+0.5,j}^{f-1}\right){\left(\frac{\partial c_{d}}{\partial z}\right)}_{i+0.5,j}^{f-1}\left.-D\left(T_{i-0.5,j}^{f-1}\right){\left(\frac{\partial c_{d}}{\partial z}\right)}_{i-0.5,j}^{f-1}\right]\right. \end{array}$$

In this case, the differential quotients are expressed as follows [17]:

(17) $$\begin{array}{l} \begin{array}{c} \begin{array}{c} {\left(rD\left(T_{i,j+0.5}^{f-1}\right)\frac{\partial c_{d}}{\partial r}\right)}_{i,j+0.5}^{f-1}=r_{i,j+0.5}\;D_{i,j+0.5}^{f-1}\frac{{\left(c_{d}\right)}_{i,j+1}^{f-1}-{\left(c_{d}\right)}_{i,j}^{f-1}}{h_{1}}=\left(r_{i,j}+\frac{h_{1}}{2}\right)\frac{{\left(c_{d}\right)}_{i,j+1}^{f-1}-{\left(c_{d}\right)}_{i,j}^{f-1}}{W_{i,j+1}^{f-1}} \end{array} \end{array}\\ \begin{array}{c} \begin{array}{c} \begin{array}{c} \begin{array}{c} {\left(rD\left(T_{i,j-0.5}^{f-1}\right)\frac{\partial c_{d}}{\partial r}\right)}_{i,j-0.5}^{f-1}=r_{i,j-0.5}\;D_{i,j-0.5}^{f-1}\frac{{\left(c_{d}\right)}_{i,j}^{f-1}-{\left(c_{d}\right)}_{i,j-1}^{f-1}}{h_{1}}=\left(r_{i,j}-\frac{h_{1}}{2}\right)\frac{{\left(c_{d}\right)}_{i,j}^{f-1}-{\left(c_{d}\right)}_{i,j-1}^{f-1}}{W_{i,j-1}^{f-1}} \end{array} \end{array} \end{array} \end{array}\\ {\left(D\left(T_{i+0.5,j}^{f-1}\right)\frac{\partial T}{\partial z}\right)}_{i+0.5,j}^{f-1}=D_{i+0.5}^{f-1}\frac{T_{i+1,j}^{f-1}-T_{i,j}^{f-1}}{h_{2}}=\frac{{\left({\bar{C}}_{d}\right)}_{i+1,j}^{f-1}-{\left({\bar{C}}_{d}\right)}_{i,j}^{f-1}}{W_{i+1,j}^{f-1}}\\ {\left(D\left(T_{i-0.5}^{f-1}\right)\frac{\partial T}{\partial z}\right)}_{i-0.5,j}^{f-1}=D_{i-0.5,j}^{f-1}\frac{T_{i,j}^{f-1}-T_{i-1,j}^{f-1}}{h_{2}}=\frac{{\left(c_{d}\right)}_{i-1,j}^{f-1}-{\left(c_{d}\right)}_{i,j}^{f-1}}{W_{i-1,j}^{f-1}} \end{array}$$

where *W* is the mass diffusion resistance.

Hence, the final formula for Equation (6) is the following [17]:

(18) $${\left(c_{d}\right)}_{i,j}^{f}={\left(c_{d}\right)}_{i,j}^{f-1}+\Delta t\sum_{a=1}^{4}\frac{\Phi_{e}}{W_{e}^{f-1}}\left[{\left(c_{d}\right)}_{e}^{f-1}-{\left(c_{d}\right)}_{i,j}^{f-1}\right]$$

where the individual *a* corresponds to *e* = {(*i*, *j* + 1); (*i*, *j* − 1); (*i* + 1, *j*); (*i* − 1, *j*)}.

The concentration at boundary nodes is deduced in a similar approach and will not be explained here—please see detailed description in literature [17].

For the explicit scheme of the finite difference method, it is necessary to specify the stability condition [17]:

(19) $$\Delta t\leq \sum_{a=1}^{4}\frac{R_{e}}{\Phi_{e}}\;\mathrm{and}\;\Delta t\leq \sum_{a=1}^{4}\frac{W_{e}^{f-1}}{\Phi_{e}}$$

### 2.3. Inverse Problem and Evolutionary Algorithm

The main goal of this study was to identify the thermophysical properties of the tissue: thermal conductivity λ [W·m^−1^·K^−1^)], density ρ [kg·m^−3^] and specific heat capacity *c* [J·kg^−1^·K^−1^] (cf. Equations (1) and (2)). Fitness function *S* was defined as follows:

(20) Sλ,ρ,c=∑f=1F∑k=1Kcdkf−cdEXPkf2→MIN

where cdkf are the concentration at the control points (selected points in the domain considered), resulting from the numerical solution of the direct problem for assumed values of λ, ρ and *c*; in turn, the cdEXPkf is a concentration based on experimental data at the control points, *K* represents the number of internal control nodes and *F* is a number of time steps. The minimum of the objective function (20) was obtained through an evolutionary algorithm, utilizing floating-point coding and own software. The evolutionary algorithm is part of the artificial intelligence and bio-inspired algorithm categories. It does not require analyzing the influence of design variables on the identification criterion but enables finding an optimal solution within an acceptable error margin [21,26].

**Figure 3:**
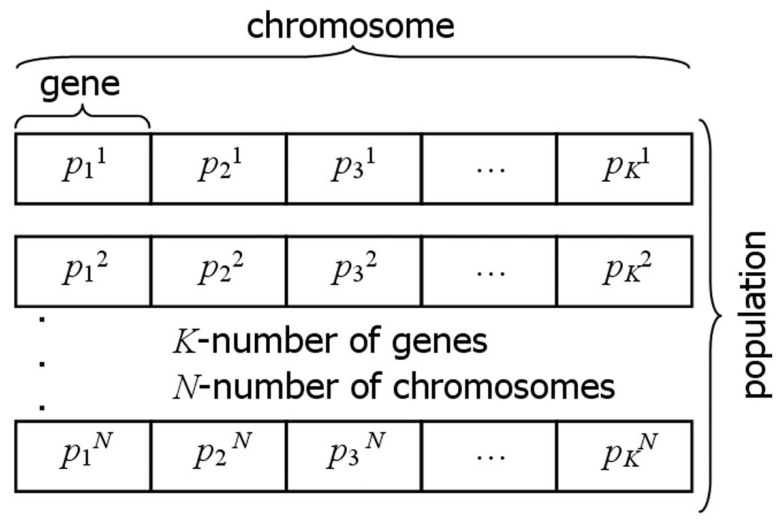
Structure of the gene, chromosome and population [21].

The evolutionary algorithm (EA) operates on the principle of modifying populations of chromosomes, which represent potential solutions. Each chromosome is made of genes, the number of which corresponds to the number of identified parameters [27,28,29]. Genes, chromosomes and population structure, for the general case, are presented in Figure 3.

The chromosome **p** contains information about the identified parameters in the following form:

(21) $$p={\left[\begin{array}{ccc} \lambda & \rho & c \end{array}\right]}^{T}$$

where λ, ρ and *c* are the genes. Gene values, representing potential solutions, are generated through operations specific to the evolutionary algorithm, taking into account the relevant constraints:

(22) $$\lambda^{L}\leq \lambda \leq \lambda^{H},{\;\rho }^{L}\leq \rho \leq \rho^{H},\;c^{L}\leq c\leq c^{H}$$

where *L* and *H* represent the minimum and maximum values of the constraints applied to the identified parameters. The limitations are collected in Equation (23).

Table 1 presents the basic parameters of the evolutionary algorithm regarding the population size, the maximum number of generations and the probability values of the evolutionary operators. The probability of occurrence of evolutionary operators was determined at the classical level for the evolutionary algorithm (cf. Table 1).

**Figure 4:**
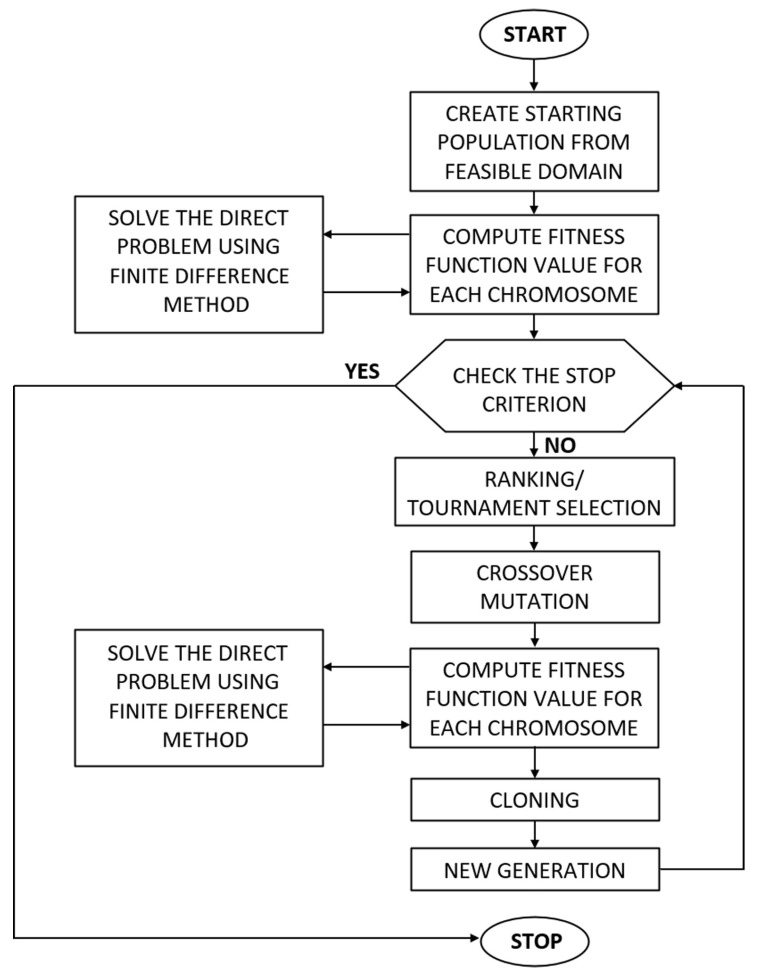
Diagram of the evolutionary algorithm workflow [21].

The evolutionary algorithm starts by generating an initial population, guided by the constraints imposed on the genes (see Equation (22)). The new population consists of *N* chromosomes **p***^n^*, *n* = 1, 2, …, *N*, randomly generated using a pseudorandom number generator with a randomly selected seed—Figure 3. Each gene value, generated during the creation of the initial population, was defined by a continuous uniform distribution. Each randomly generated gene value falls within the allowable domain. For the given **p***^n^* values, the direct problem was solved using the numerical method presented in Section 2.1 and Section 2.2. Next, the fitness (objective) function (20) is evaluated for each chromosome **p***^n^*, the stopping criterion is checked, and if it is not satisfied, the selection operator is applied. Selection is performed through either ranking selection or tournament selection (with equal probability). Based on the selection process, some chromosomes undergo crossover and mutation operators’ application according to the probabilities specified in Table 1. The next step of the EA involves calculating the fitness function (20) for the modified individuals, followed by the application of the cloning operator and, finally, the creation of a new generation of chromosomes. The flow chart of evolutionary algorithm is presented in Figure 4. The stopping criteria for the evolutionary algorithm are implemented on the following criteria [21,27,29]:

- The fitness function value is zero.
- Once the predefined number of generations is reached.
- Insufficient improvement in the fitness function value across successive iterations.

During the operation of the evolutionary algorithm, the following evolutionary operators were used:

- A uniform mutation operator that modifies the gene values in a chromosome by randomly selecting new ones.
- A non-uniform mutation operator that uses a Gaussian distribution to change the gene values in a chromosome. The amplitude of such mutation in each generation is equal to $\sigma_{Gauss}=1/pop$, where *pop* is the number of generations.

- An arithmetic crossover operator that creates a new chromosome by forming a linear combination of genes from two randomly selected chromosomes, a cloning that enables the best chromosome to be carried over to the next population.

Unlike genetic algorithms, evolutionary algorithms allow the probability of the mutation operator to vary with each generation. In this case, the mutation probability remains constant, but the mutation strength decreases through a non-uniform mutation based on a Gaussian distribution.

The evolutionary algorithm used to solve the inverse problem was created in the Delphi environment by the authors of this article.

## 3. Results and Discussion

The cryopreservation process, consisting in storing biological material at low temperatures, involves several complex physical phenomena. Numerical modelling makes it possible to understand and analyze processes such as heat and mass transport, phase transformations and others. Numerical modelling can be used to optimize the conditions under which cryopreservation is carried out to increase cell survival by minimizing the damage caused by ice crystallization. Numerical modelling is an extremely important part of modern cryopreservation research.

Solving the direct problem, the distributions of temperature and DMSO concentration in the extracellular matrix during the cooling process were obtained. Bath solution parameters were regulated according to the LT protocol developed by Pegg et al. [18], which included eight steps in the cooling phase, to which this work was limited. Then, the calculations for the inverse problem were performed to identify the thermophysical parameters based on the experimental results.

### 3.1. Direct Problem

The direct problem presents a study of a homogeneous sample of articular cartilage with the dimensions *R* = 3 mm and *H* = 1 mm [11]. To perform the thermal analysis, the following parameters were introduced: α = 525 W·m^−2^·K^−1^ and *T*_0_ = 22 °C [11]. For the mass transfer model, it is necessary to know the chemical properties of the CPA (DMSO solution in water), such as *r_s_* = 2.541·10^−10^ m [30], μ = 1.996·10^−3^ Pa·s [31] and initial value *c*_0_ = 0% (*w*/*w*) [11].

The bath solution parameters were specified in accordance with the LT protocol developed by Pegg et al. [18]. This protocol included eight steps in the cooling phase and seven steps in the warming phase, but the article is limited to the cooling phase only. The values of *T_bath_* and *c_bath_*, which change at each step of the process, are detailed in Table 2.

Numerical simulations were performed in an author’s program using FDM as described in Section 2.2. The time and mesh steps were assumed to be as follows: ∆*t* = 0.005 s, *h*_1_ = 0.0001 m and *h*_2_ = 0.00005 m.

Firstly, a direct problem was calculated, for which we introduced the thermophysical parameters of the articular cartilage taken from the literature, where *c* = 3567.5 J·kg^−1^·K^−1^, ρ = 1100 kg·m^−3^ and λ = 0.518 W·m^−1^·K^−1^ [32,33]. Then, the results obtained were compared with those obtained by solving the inverse problem.

**Figure 5:**
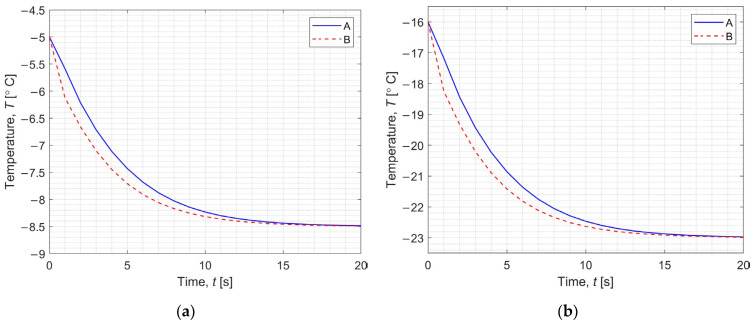
Temperature as a function of time in the first 20 s of step 4 (**a**) and step 6 (**b**).

Figure 5 shows the temperature change during the first 20 s of step 4 (a) and step 6 (b) for points A (*r* = 0.00005 m, *z* = 0.000475 m, solid line) and B (*r* = 0.00145 m, *z* = 0.000025 m, dashed line). From this graph, it can be seen that the temperature in the domain stabilizes relatively quickly with respect to the duration of the entire particular step (it is about 40 s for step 4 and 6).

**Figure 6:**
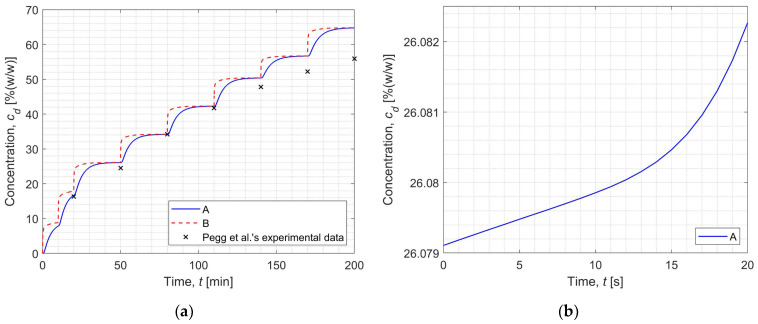
Concentration of DMSO as a function of time during the entire freezing phase (the ‘x’ points are from [18]) (**a**) and in the first 20 s of step 4 (**b**).

On the other hand, Figure 6 depicts the change in DMSO concentration (CPA) in the extracellular matrix at points A (solid line) and B (dashed line) during the entire freezing phase of the cryopreservation process (cf. Figure 6a) and for the first 20 s of step 4 (cf. Figure 6b). Figure 6a additionally provides points corresponding to experimental data from the literature [18].

**Figure 7:**
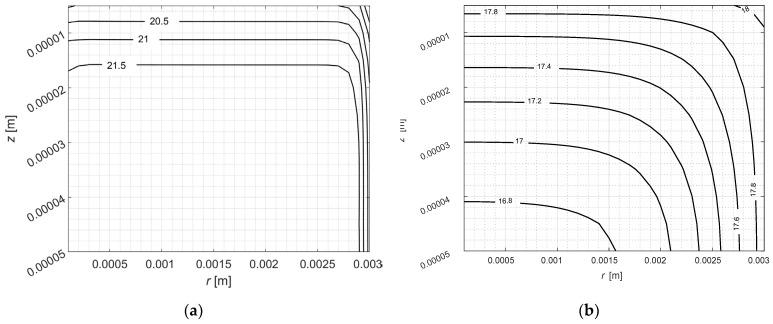
Distribution of temperature (**a**) and concentration of DMSO (**b**) in computational domain after 10 s of step 3.

Figure 7 illustrates the distribution of temperature (a) and DMSO concentration (b) in the computational domain of the sample after 10 s of step 3 (in 1210 s of the whole process). These distributions also indicate that the analyzed problem is axisymmetric.

In addition, Table 3 contains the exact values of temperature and DMSO concentration in the extracellular matrix at the end of each step of the freezing phase in point A compared with the experimental results [18]. Table 3 also includes the calculated relative error values of the obtained simulation results with respect to the experimental measurements from the literature.

By comparing the graph in Figure 6a and the values in Table 3, it can be noted that there are discrepancies between the experimental and simulation results. One can observe that the lowest relative error is 0.06% at the end of step 4, from which it increases continuously until it reaches the highest value—15.83% (end of step 8, end of simulation). Similar conclusions were discussed in previous papers [16,17]. The literature [16,17] indicates that these discrepancies may be caused by the relation used to determine the diffusion coefficient. This paper applies the Einstein–Stokes equation (Equation (7)) to calculate the diffusion coefficient, while it can be found in the literature that this variable is estimated from other relationships, considering the tissue as a porous material (for example, in [11]).

On the other hand, these incompatibilities may be caused by the introduced thermophysical parameters. It can be said that these variables are uncertain, because their values are determined experimentally and are dependent on many factors, e.g., the quality of the collected samples to research or the condition of the tested organism. Therefore, an identification of the thermophysical parameters of the articular cartilage was carried out to verify that the variables selected from the literature do not introduce computational errors. The results of the inverse problem are presented in the next section.

### 3.2. Inverse Problem

Using the databases of the Foundation for Research on Information Technologies in Society [33], it was found that the ranges of values of the identified parameters for articular cartilage are within the following intervals:

- Thermal conductivity λ = 0.47 ÷ 0.52 W·m^−1^·K^−1^.
- Specific heat capacity *c* = 3500 ÷ 3664 J·kg^−1^·K^−1^.
- Density ρ = 1099 ÷ 1100 kg·m^−3^.

In view of the above, for the purpose of the calculations, it was assumed that the constraints for the identified parameters fall within the following ranges:

(23) $$\begin{array}{c} 0.47\leq \lambda \leq 0.52\\ 3500\leq c\leq 3700\\ 1050\leq \rho \leq 1150 \end{array}$$

As can be seen in Figure 6a and Table 3, the results obtained by numerical simulation using the finite difference method (own code) and the experimental data taken from the literature [18] are very similar to each other and involve only one computational step (step 8) error greater than 10%. Based on this information, three thermophysical parameters of articular cartilage were identified using an evolutionary algorithm. Two cases were analyzed; in the first one (“case 1”), information on DMSO concentration from all eight steps was used to calculate the fitness function (22) of each chromosome (cf. Table 3), and in the second one (“case 2”), information on the DMSO concentration from seven steps was used (cf. Table 3), discarding the result with the largest error. In the Table 4, Table 5, Table 6 and Table 7 the inverse problem solutions and comparison of DMSO concentration with experimental data are presented for different cases. Identification results for the described cases are presented in Table 4 and Table 6. In turn, the DMSO concentration values for the identified parameters are compared to the experiment in Table 5 and Table 7, for cases “1” and “2”, respectively.

**Figure 8:**
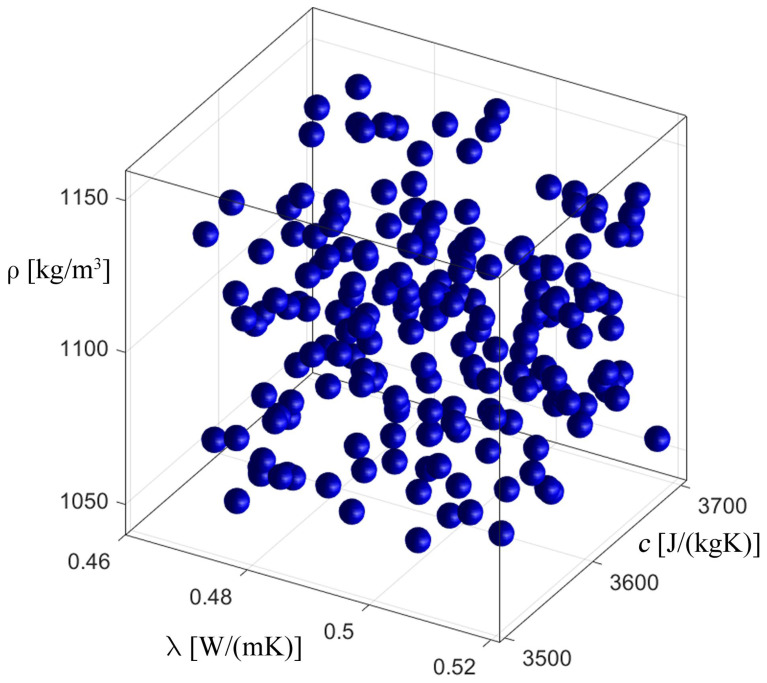
The process of identification—evolutionary algorithm—after 1st generation (“case 1”).

**Figure 9:**
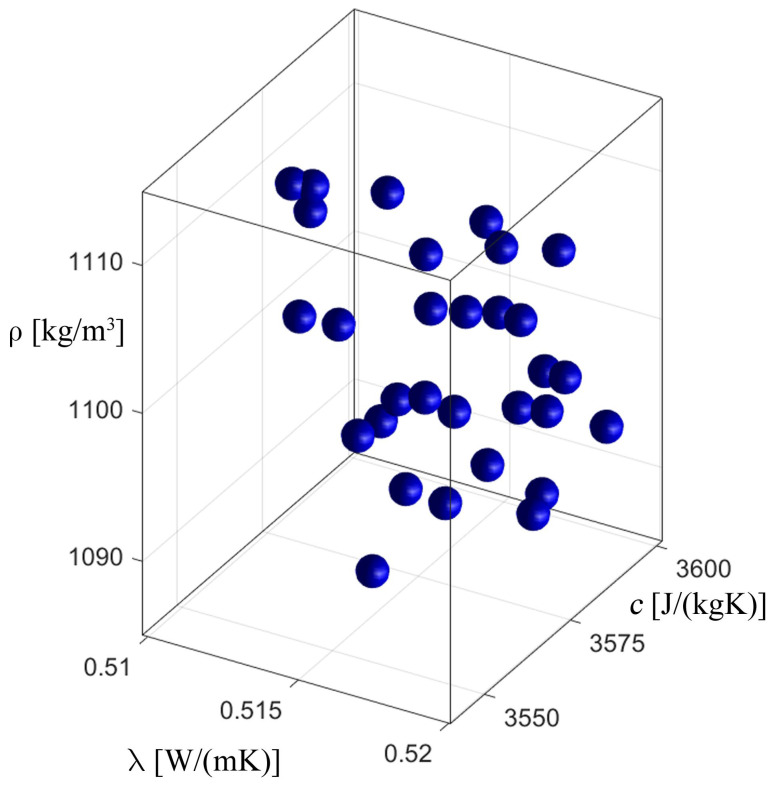
The process of identification—evolutionary algorithm—after 300th generation (“case 1”).

**Figure 10:**
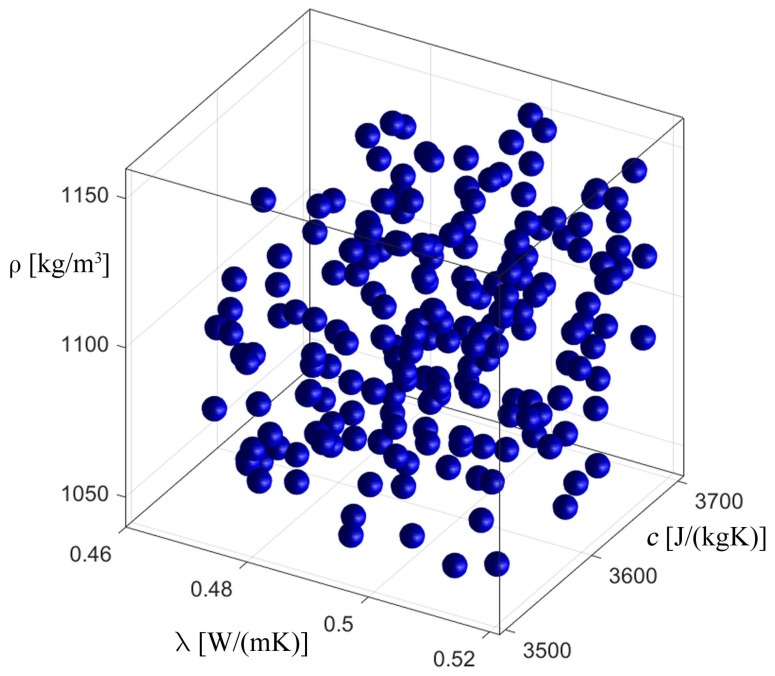
The process of identification—evolutionary algorithm—after 1st generation (“case 2”).

**Figure 11:**
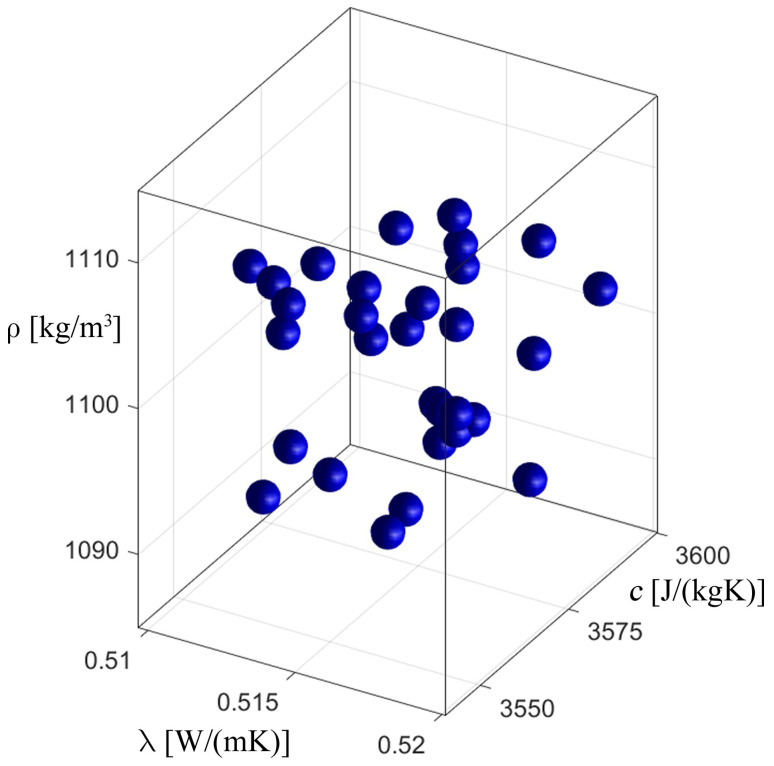
The process of identification—evolutionary algorithm—after 300th generation (“case 2”).

Figure 8, Figure 9, Figure 10 and Figure 11 show the process of identification using an evolutionary algorithm after the 1st (1st generation means it is an initial population) and 300th generations (last generation) for “case 1” (cf. Figure 8 and Figure 9) and for “case 2” (cf. Figure 10 and Figure 11). As can be seen, for both cases, the starting population covers the entire range of possible values of the identified parameters, consistent with the constraints (23), and, in turn, in the last generation the individuals cluster primarily around the optimum, which is consistent with the specific operation of the evolutionary algorithm. Figure 9 and Figure 11 show chromosomes of the last generation, only near the optimal solution. The stopping criterion for the evolutionary algorithm was set to the maximum number of generations. Observing the value of the fitness function (20) of the best chromosome, while the evolutionary algorithm is running, allows one to conclude that its value has not changed in recent generations.

## 4. Conclusions

The direct problem was formulated as an axisymmetric heat and mass transfer model, complemented by a liquidus tracking method protocol. This protocol adjusts the temperature and concentration to ensure that the temperature of the sample remains above or on the liquidus line, thereby eliminating the likelihood of ice crystallization in the cells.

The obtained results of the temperature and DMSO concentrations were compared with experimental results taken from the literature, which were obtained by the research team of Pegg et al. [18]. The temperature distribution in the sample area relatively quickly reached values according to the established LT protocol. However, it was noted that there are discrepancies between the numerical simulation results and the experimental data. We believe that these incompatibilities may be caused by the assumed thermophysical parameters.

The literature reports experimentally determined values for these parameters, which differ according to the source. In fact, the thermophysical parameters of biological samples depend on many different factors, such as age, sex, etc. [16,17,34]. Therefore, in some papers, thermophysical parameters are considered as uncertain parameters and are defined, for example, as interval numbers or fuzzy numbers to account for these imprecisions in their estimation [16,23,35,36].

The aim of this study was to identify the thermophysical parameters of articular cartilage during the cryopreservation process, which are crucial for preparing a proper mathematical model to simulate cryopreservation. Three parameters were the subject of identification, namely thermal conductivity, specific heat capacity and density. This article proposes a novel concept to solve the identification problem (inverse problem) to examine whether the applied parameters are not the reason for the calculation error or to confirm that the data adopted from the literature are correct.

The number of individuals in the population of an evolutionary algorithm directly affects the quality of the solution and the computation time. A larger population provides a greater diversity of individuals, which increases the chances of finding a better solution, but may lead to a longer computation time. A smaller population may converge to the solution faster but with a greater risk of getting stuck in a local minimum. In turn, a larger population increases computational costs because it requires the evaluation of a larger number of individuals in each generation. A smaller population shortens the computation time but may not provide sufficient diversity, which limits the quality of the solution. The authors’ many years of experience in working with evolutionary algorithms allowed them to choose the population size so as to maintain an appropriate balance between the quality of the solution and the computation time. The population size is given in Table 1.

During the operation of the evolutionary algorithm, two types of selection were used to choose individuals that would be subjected to the evolutionary operators: tournament selection and ranking selection. During its operation, the algorithm randomly chose which selection method would be applied in each generation, based on an equal probability of occurrence. In the case of tournament selection, it involved randomly selecting individuals who “compete” against each other, with the winner (the best individual) advancing to the pool of individuals that will undergo crossover or mutation operators’ application. This type of selection promotes the preservation of population diversity, while giving preference to the best individuals. In the case of ranking selection, individuals are sorted based on the value of the fitness function and then selected proportionally to their quality. By ensuring that individuals with similar fitness function value have comparable chances of passing to the next generation, this selection method helps maintain greater diversity within the population. Additionally, in ranking selection, there is less emphasis on the best individuals compared to tournament selection.

Two types of mutations were used in the algorithm: uniform and non-uniform (Gaussian) mutations. The first type changes the gene value to a random value from a specified range (cf. Equation (23)), thus allowing a high diversity of individuals and aiding in the exploration of the solution space. However, its effect is limited by a low probability of occurrence (cf. Table 1). On the other hand, the non-uniform (Gaussian) mutation changes the gene value by adding a random value from a normal distribution, causing a “gentle” change in the gene value (with the change becoming smaller as the generation number increases). This mutation supports the accuracy of the solutions and effectively explores the space around the best solutions.

The evolutionary algorithm, being part of the group of metaheuristic algorithms, can have numerous modifications due to the variety of approaches to its operation. One such element is the stopping criterion for the calculations. Usually, a single criterion is used, but there is no obstacle to combining them. The most commonly used criteria include: reaching the maximum number of generations—the algorithm terminates after a specified number of generations, regardless of the quality of the solution; no improvement in the objective function—when there is no significant improvement in the solution over several consecutive generations, the algorithm can be stopped; reaching a specified objective function value—the algorithm stops when the objective function reaches a value considered sufficient; population convergence—when the population becomes homogeneous, meaning most individuals have similar solutions, which may indicate that the optimal solution has been reached. As stated in Section 3.2, the maximum number of generations was applied.

Analyzing the results obtained from solving the inverse problem, it can be assumed that the parameters available in the literature (cf. Section 3.1) are correct (cf. Table 6). Therefore, it is worth considering the differences in the results obtained from the numerical simulation and the experiment data (cf. Figure 6a, Table 3) and checking which of the parameters of the mathematical model has a significant impact on the result of the numerical simulation. Sensitivity analysis methods can undoubtedly help to solve this problem, and the topic will certainly be discussed by the authors in future scientific research.

In conclusion, the solution to the inverse problem considered in this paper is very satisfying.

An undeniable advantage of the evolutionary algorithm is its universality, as it only processes information about the value of the objective function and, based on this, searches for the optimal solution. One could argue that a properly designed evolutionary algorithm only requires information about the solution to the direct problem, obtained using any numerical tool, whether it is one’s own code or results from commercial software. This approach was chosen due to authors’ many years of experience in developing proprietary algorithms based on evolutionary theory. In the case of classical optimization algorithms, which the authors also worked on (i.e., gradient-based methods), it would be necessary to differentiate the mathematical model with respect to the identified parameters, which, due to the complex mathematical description, would be a highly complicated task. In the future, the authors plan to attempt the use of sensitivity analysis methods (where sensitivity functions are also utilized in gradient algorithms) to determine the impact of mathematical model parameters on the obtained calculation results. Among other optimization algorithms, it is certainly worth mentioning machine learning, which is currently very popular and one of the most rapidly evolving areas in technology and science. However, the authors have no experience in this area.

Finally, it is worth mentioning some limitations of this work. The model was developed for the selected tissue and CPAs—articular cartilage and DMSO—which were applied in the experimental research presented by Pegg et al. [18]. However, it should be remembered that the cryopreservation process can be executed for different types of biological tissues, for example, oocytes and embryos, sperm, semen and testicular tissue, stem cells or hepatocytes and using different CPAs, such as glycol [1]. Meanwhile, our developed model is quite universal, because a change in biological tissue and CPA will result in a modification of input parameters only, such as thermophysical parameters. If the tissue in question contains blood and lymphatic vessels, the Fourier equation should be supplemented additionally with the heat source *Q* (compare with Equation (1)).

As a limitation, it can also be considered that the validation of the results was achieved only by comparing with data from the literature. The authors’ lack of conducting their own experiments is mainly due to the difficulties related to research on biological tissues, including the problem of obtaining samples and access to appropriate medical equipment. However, the modelling of cryopreservation has practical applications. A well-prepared model can later be used for osmotic transport analysis, which allows one to estimate changes in the cell volume of a given tissue and, in effect, to assess the damage of them. Moreover, understanding the changes that occur in cells during the process gives the opportunity to modify the LT protocol to expose the selected tissue less to the toxic impact of CPA, without the need for another experiment (compare with the approach presented by Yu et al. [11]). In addition, both finite difference and evolutionary algorithms have limitations that can affect the accuracy and reliability of the results obtained. In the case of the finite difference method, there may be a problem in obtaining adequate accuracy for areas with large gradients and in discretizing the area. Problems with evolutionary algorithms include sensitivity to the choice of parameters such as population size, mutation and crossover rates and long simulation times. The use of hybrid algorithms, for example, can minimize these errors and improve the computational efficiency of both methods.

Solving the direct problem using a numerical algorithm based on the finite difference method enabled the simulation of the cryopreservation process through mathematical modelling. The results obtained from the numerical analysis show a high degree of consistency with the experimental results. The solution of the inverse problem aimed at identifying the thermophysical parameters of the analyzed object, specifically targeting the values for articular cartilage. As indicated, these parameters fall within wide ranges due to the individual characteristics of biological tissues. The results obtained suggest that both the direct and inverse analysis processes were conducted accurately. Future work will focus on refining the mathematical model to achieve results even closer to experimental outcomes.

In further scientific work, the authors would like to conduct similar types of analyses for other cryoprotectants, as well as simulate cryopreservation for other biological tissues performed by a different method. It will also be the subject of further research to use hybrid methods to solve the problem presented in this paper.

## Figures and Tables

**Table 1:** Evolutionary algorithm parameters.

Parameter	Value
Number of generations	300
Number of chromosomes	200
Probability of uniform mutation	5%
Probability of non-uniform mutation	5%
Probability of arithmetic crossover	40%
Probability of cloning	100%

**Table 2:** LT protocol for cooling phase.

Step	Time Duration	Temperature of Bath Solution	Concentration of Bath Solution
	*t* [min]	*T_bath_* [°C]	*c_bath_* [%(*w*/*w*)]
1	10	22	10
2	10	22	20
3	30	−5	29
4	30	−8.5	38
5	30	−16	47
6	30	−23	56
7	30	−35	63
8	30	−48.5	72

**Table 3:** Comparison of DMSO concentration with experimental data.

Step	DMSO Concentration	Experimental Data	Relative Error
	*c_d_* [%(*w*/*w*)]	*c_d_* [%(*w*/*w*)]	δ [%]
1	7.8411	-	-
2	16.7257	16.3 ± 1.3	2.6118
3	26.0791	24.5 ± 1.1	6.4453
4	34.1795	34.2 ± 0.9	0.0600
5	42.2754	41.7 ± 3.3	1.3799
6	50.3709	47.8 ± 2.8	5.3784
7	56.6698	52.2 ± 1.3	8.5628
8	64.7463	55.9 ± 2.9	15.8253

**Table 4:** Inverse problem solution using evolutionary algorithm for “case 1”.

Parameter	Value from [32,33]	Found Value	Fitness Function
Thermal conductivity, λ [W·m^−1^·K^−1^]	0.518	0.522	107.852194
Specific heat capacity, *c* [J·kg^−1^·K^−1^]	3567.5	3571.2	
Density, ρ [kg·m^−3^]	1100	1089.3	

**Table 5:** Comparison of DMSO concentration with experimental data and numerical solution based on identified thermophysical parameters using evolutionary algorithm for “case 1”.

Step	DMSO Concentration	Experimental Data
	*c_d_* [%(*w*/*w*)]	*c_d_* [%(*w*/*w*)]
1	7.8411	-
2	16.7257	16.3 ± 1.3
3	26.0791	24.5 ± 1.1
4	34.1794	34.2 ± 0.9
5	42.2754	41.7 ± 3.3
6	50.3708	47.8 ± 2.8
7	56.6697	52.2 ± 1.3
8	64.7463	55.9 ± 2.9

**Table 6:** Inverse problem solution using evolutionary algorithm for “case 2”.

Parameter	Value from [32,33]	Found Value	Fitness Function
Thermal conductivity, λ [W·m^−1^·K^−1^]	0.518	0.519	29.594674
Specific heat capacity, *c* [J·kg^−1^·K^−1^]	3567.5	3565.9	
Density, ρ [kg·m^−3^]	1100	1100	

**Table 7:** Comparison of DMSO concentration with experimental data and numerical solution based on identified thermophysical parameters using evolutionary algorithm for “case 2”.

Step	DMSO Concentration	Experimental Data
	*c_d_* [%(*w*/*w*)]	*c_d_* [%(*w*/*w*)]
1	7.8411	-
2	16.7257	16.3 ± 1.3
3	26.0791	24.5 ± 1.1
4	34.1794	34.2 ± 0.9
5	42.2754	41.7 ± 3.3
6	50.3708	47.8 ± 2.8
7	56.6697	52.2 ± 1.3
8	64.7463	55.9 ± 2.9

## Data Availability

The original contributions presented in the study are included in the article, further inquiries can be directed to the corresponding author.

## References

[1] Jang T.H., Park S.C., Yang J.H., Kim J.Y., Seok J.H., Park U.S., Choi C.W., Lee S.R., Han J. (2017). Cryopreservation and Its Clinical Applications. Integr. Med. Res..

[2] Xu F., Moon S., Zhang X., Shao L., Song Y.S., Demirci U. (2010). Multi-Scale Heat and Mass Transfer Modelling of Cell and Tissue Cryopreservation. Philos. Trans. R. Soc. A Math. Phys. Eng. Sci..

[3] Jungare K.A., Radha R., Sreekanth D. (2022). Cryopreservation of Biological Samples—A Short Review. Mater. Today Proc..

[4] Kulkarni P.G., Paudel N., Magar S., Santilli M.F., Kashyap S., Baranwal A.K., Zamboni P., Vasavada P., Katiyar A., Singh A.V. (2024). Overcoming Challenges and Innovations in Orthopedic Prosthesis Design: An Interdisciplinary Perspective. Biomed. Mater. Devices.

[5] Trzcińska M., Bryła M. (2020). Kierunki i Możliwości Modyfikacji Metod Kriokonserwacji Oraz Oceny Jakości Nasienia Knura.

[6] Mazur P. (1963). Kinetics of Water Loss from Cells at Subzero Temperatures and the Likelihood of Intracellular Freezing. J. Gen. Physiol..

[7] Kay A.G., Hoyland J.A., Rooney P., Kearney J.N., Pegg D.E. (2015). A Liquidus Tracking Approach to the Cryopreservation of Human Cartilage Allografts. Cryobiology.

[8] Singh S., Kumar S. (2015). Freezing of Biological Tissues During Cryosurgery Using Hyperbolic Heat Conduction Model. Math. Model. Anal..

[9] Wang Z., Zhao G., Wang T., Yu Q., Su M., He X. (2015). Three-Dimensional Numerical Simulation of the Effects of Fractal Vascular Trees on Tissue Temperature and Intracelluar Ice Formation during Combined Cancer Therapy of Cryosurgery and Hyperthermia. Appl. Therm. Eng..

[10] Kumar S., Singh S., Mondaini R.P. (2018). Numerical Study on Biological Tissue Freezing Using Dual Phase Lag Bio-Heat Equation. Trends in Biomathematics: Modeling, Optimization and Computational Problems: Selected Works from the BIOMAT Consortium Lectures, Moscow 2017.

[11] Yu X., Zhang S., Chen G. (2019). Modeling the Addition/Removal of Dimethyl Sulfoxide into/from Articular Cartilage Treated with the Liquidus-Tracking Method. Int. J. Heat Mass Transf..

[12] Zhou X., Jiang Z., Liang X.M., Liu J., Fang P., Liu Z., Gao D. (2020). Microfiltration-Based Sequential Perfusion: A New Approach for Improved Loading/Unloading of Cryoprotectants. Sens. Actuators B Chem..

[13] Liu W., Zhao G., Shu Z., Wang T., Zhu K., Gao D. (2015). High-Precision Approach Based on Microfluidic Perfusion Chamber for Quantitative Analysis of Biophysical Properties of Cell Membrane. Int. J. Heat Mass Transf..

[14] Zhang S., Yu X., Chen G. (2012). Permeation of Dimethyl Sulfoxide into Articular Cartilage at Subzero Temperatures. J. Zhejiang Univ. Sci. B.

[15] Elmoazzen H.Y., Elliott J.A.W., McGann L.E. (2009). Osmotic Transport across Cell Membranes in Nondilute Solutions: A New Nondilute Solute Transport Equation. Biophys. J..

[16] Skorupa A., Piasecka-Belkhayat A. (2020). Numerical Modeling of Heat and Mass Transfer during Cryopreservation Using Interval Analysis. Appl. Sci..

[17] Skorupa A. (2023). Multi-Scale Modelling of Heat and Mass Transfer in Tissues and Cells During Cryopreservation Including Interval Methods. Ph.D. Thesis.

[18] Pegg D.E., Wang L., Vaughan D. (2006). Cryopreservation of Articular Cartilage. Part 3: The Liquidus-Tracking Method. Cryobiology.

[19] Paruch M. (2018). Identification of the Degree of Tumor Destruction on the Basis of the Arrhenius Integral Using the Evolutionary Algorithm. Int. J. Therm. Sci..

[20] Mochnacki B., Majchrzak E., Paruch M. (2018). Soft Tissue Freezing Process. Identification of the Dual-Phase Lag Model Parameters Using the Evolutionary Algorithm. AIP Conf. Proc..

[21] Paruch M., Piasecka-Belkhayat A., Korczak A. (2023). Identification of the Ultra-Short Laser Parameters during Irradiation of Thin Metal Films Using the Interval Lattice Boltzmann Method and Evolutionary Algorithm. Adv. Eng. Softw..

[22] Fourier J.B.J. (1882). Théorie Analytique de La Chaleur.

[23] Piasecka-Belkhayat A., Skorupa A. (2022). Application of Interval Arithmetic in Numerical Modeling of Cryopreservation Process during Cryoprotectant Loading to Microchamber. Numer. Heat Transf. Part A Appl..

[24] Majchrzak E., Mochnacki B. (2004). Numerical Methods. Theoretical Base, Practical Aspects, Algorithms.

[25] Mochnacki B., Suchy J. (1995). Numerical Methods in Computations of Foundry Processes.

[26] Singh A.V., Chandrasekar V., Prabhu V.M., Bhadra J., Laux P., Bhardwaj P., Al-Ansari A.A., Aboumarzouk O.M., Luch A., Dakua S.P. (2024). Sustainable Bioinspired Materials for Regenerative Medicine: Balancing Toxicology, Environmental Impact, and Ethical Considerations. Biomed. Mater..

[27] Arabas J. (2001). Lectures of Evolutionary Algorithms.

[28] Paruch M., Majchrzak E. (2007). Identification of Tumor Region Parameters Using Evolutionary Algorithm and Multiple Reciprocity Boundary Element Method. Eng. Appl. Artif. Intell..

[29] Michalewicz Z. (1996). Genetic Algorithms + Data Structures = Evolution Programs.

[30] Schulze B.M., Watkins D.L., Zhang J., Ghiviriga I., Castellano R.K. (2014). Estimating the Shape and Size of Supramolecular Assemblies by Variable Temperature Diffusion Ordered Spectroscopy. Org. Biomol. Chem..

[31] https://www.trimen.pl/witek/ciecze/old_index.html.

[32] Youn J.-I., Telenkov S.A., Kim E., Bhavaraju N.C., Wong B.J.F., Valvano J.W., Milner T.E. (2000). Optical and Thermal Properties of Nasal Septal Cartilage. Lasers Surg. Med..

[33] https://itis.swiss/virtual-population/tissue-properties/database/.

[34] Mochnacki B., Piasecka-Belkhayat A. (2013). Numerical Modeling of Skin Tissue Heating Using the Interval Finite Difference Method. Mol. Cell. Biomech..

[35] Piasecka-Belkhayat A., Skorupa A. (2024). Cryopreservation Analysis Considering Degree of Crystallisation Using Fuzzy Arithmetic. J. Theor. Appl. Mech..

[36] Skorupa A., Piasecka-Belkhayat A. (2023). Comparison of Heat Transfer Phenomena for Two Different Cryopreservation Methods: Slow Freezing and Vitrification. J. Appl. Math. Comput. Mech..

